# Cytotoxic Effects of Fucoidan Nanoparticles against Osteosarcoma

**DOI:** 10.3390/md11114267

**Published:** 2013-10-30

**Authors:** Ryuichiro Kimura, Takayoshi Rokkaku, Shinji Takeda, Masachika Senba, Naoki Mori

**Affiliations:** 1 Department of Microbiology and Oncology, Graduate School of Medicine, University of the Ryukyus, 207 Uehara, Nishihara, Okinawa 903-0215, Japan; E-Mail: kimuraryuichiro@yahoo.co.jp; 2 Transdisciplinary Research Organization for Subtropics and Island Studies, University of the Ryukyus, 1 Senbaru, Nishihara, Okinawa 903-0213, Japan; 3 Department of Orthopedic Surgery, Graduate School of Medicine, University of the Ryukyus, 207 Uehara, Nishihara, Okinawa 903-0215, Japan; E-Mail: koushoujp@yahoo.co.jp; 4 Kanehide Bio Co., Ltd., 5-2-2 Nishizaki-cho, Itoman, Okinawa 901-0305, Japan; E-Mail: s-takeda.bi@kanehide.co.jp; 5 Department of Pathology, Institute of Tropical Medicine, Nagasaki University, 1-12-4 Sakamoto, Nagasaki 852-8523, Japan; E-Mail: mikiyo@nagasaki-u.ac.jp

**Keywords:** fucoidan, nanoparticle, osteosarcoma, apoptosis, lung metastasis

## Abstract

In this study, we analyzed the size-dependent bioactivities of fucoidan by comparing the cytotoxic effects of native fucoidan and fucoidan lipid nanoparticles on osteosarcoma *in vitro* and *in vivo*. *In vitro* experiments indicated that nanoparticle fucoidan induced apoptosis of an osteosarcoma cell line more efficiently than native fucoidan. The more potent effects of nanoparticle fucoidan, relative to native fucoidan, were confirmed *in vivo* using a xenograft osteosarcoma model. Caco-2 cell transport studies showed that permeation of nanoparticle fucoidan was higher than native fucoidan. The higher bioactivity and superior bioavailability of nanoparticle fucoidan could potentially be utilized to develop novel therapies for osteosarcoma.

## 1. Introduction

Fucoidan, commonly found in brown seaweeds, consists mainly of fucose and sulfate with small amounts of galactose, xylose, mannose and uronic acid [1,2]. Fucoidan has steadily attracted attention in the last two decades and is currently known to have anticoagulant, antiviral, anti-inflammatory and anticancer activities [1,2]. Our laboratory has focused on the evaluation of fucoidan for the treatment of malignancies and/or viral infectious diseases [3,4,5]. The main mechanism of the anticancer activities of fucoidan is considered to be the regulation of molecules related to apoptosis and cell cycle [6]. One main feature of fucoidan is its high molecular weight. It cannot be degraded by human digestive enzymes [7]. Several groups have attempted to develop a method to produce a low molecular weight fucoidan (LMWF) [1]. These attempts are based on the *in vitro* finding that the anticancer activity of fucoidan in lung cancer cell line is significantly improved after lowering its molecular weight [8]. Paradoxically, high molecular weight fucoidan (HMWF) promotes a greater increase in the proportion of murine cytotoxic T cells than middle or LMWF in mice fed experimental diet [9]. Nevertheless, a recent study using a colon cancer-bearing mouse model demonstrated that oral administration of LMWF increased survival time, similar to HMWF, compared with the control [10]. Thus, there is no definite agreement on the effects of molecular weight of fucoidan on its anticancer properties.

The oral route is the conventional mean of drug administration, especially in patients requiring long-term treatment. The nanoparticle approach has been recently explored for natural products and chemotherapeutic agents [11]. Biocompatible nanoparticles have been developed, which are essentially inert systemic carriers used to deliver therapeutic compounds to target cells and tissues [11]. Based on their peculiar size, nanoparticle drugs easily penetrate the cell and cell organelles; and, because of their large surface area and enhanced bioavailability, tend to be more active than their microstructure counterparts.

Osteosarcoma is the most common primary bone tumor. The peak incidence of this aggressive tumor coincides with the period of rapid skeletal growth, thus affecting mostly children and adolescents [12]. The recent introduction of neoadjuvant and adjuvant chemotherapy combined with surgery has increased the 5-year survival rate for localized disease by greater than 60% compared with surgery alone [13,14]. However, patients with advanced diseases with metastasis continue to have poor prognosis with 5-year survival rates below 30% [14]. Moreover, there are currently no effective therapeutic options for patients who relapse following administration of chemotherapeutic agents or those who suffer toxicities from chemotherapy. Therefore, the discovery and development of novel chemotherapeutic agents that can improve the survival rates of patients with osteosarcoma and/or lower the occurrence of the toxic side effects of currently approved agents is of utmost importance.

Although several reports have described the potent anti-neoplastic activity of fucoidan against several diverse types of malignancies, size-dependent bioactivities have attracted attention [2,8,9,10]. In the present study, fucoidan extracted from *Cladosiphon okamuranus* was encapsulated in nanoparticles using liposomes as nanocarriers, and its anticancer effects were assessed in a cell culture system. Furthermore, we examined the effects of oral administration of fucoidan on tumor growth and lung metastasis using an ostersarcoma tumor-bearing mouse model.

## 2. Results and Discussion

### 2.1. Cytotoxic Effects of Fucoidan on Osteosarcoma Cells

**Figure 1:**
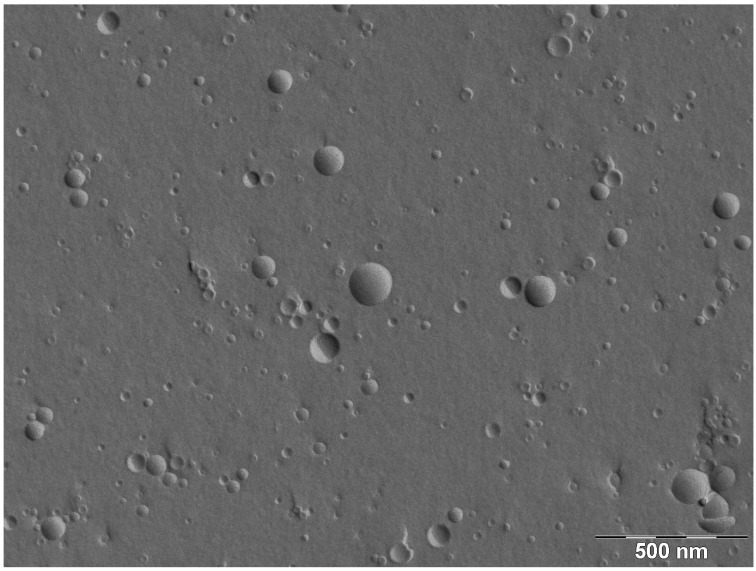
Freeze fracture electron micrograph of SLP-PC70 liposomes prepared by the mechanochemical method.

The freeze fracture electron micrography (FFEM) was undertaken to determine the structure of the obtained SLP-PC70 liposomes. Figure 1 shows a typical FFEM image of the SLP-PC70 liposomes, confirming that they are small unilamellar vesicles. The particle size of the liposomes was about 100 nm.

**Figure 2:**
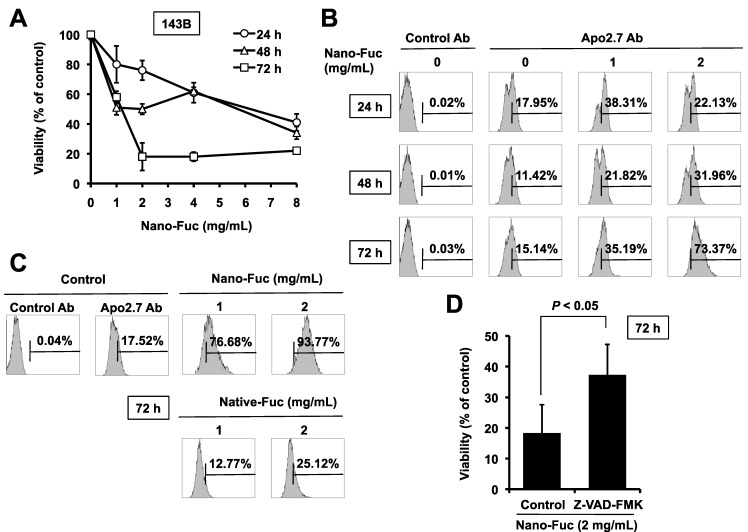
Fucoidan induces apoptosis of 143B cells, (**A**) Dose- and time-dependent reduction of cell viability in 143B cells following the addition of nanoparticle fucoidan to the cell cultures. 143B cells were treated with increasing concentrations of nanoparticle fucoidan. Twenty-four, 48 and 72 h later, cell viability was analyzed using the WST-8 assay. Data represent percentage of cell survival compared with the control and are expressed as mean ± SD (*n* = 3); (**B**) Flow cytometric analysis using Apo2.7 staining on nanoparticle fucoidan-treated and untreated cells. The analysis confirmed nanoparticle fucoidan-induced decline in 143B cell viability was the result of apoptosis; (**C**) Nanoparticle fucoidan is more potent than native fucoidan in inducing apoptosis. 143B cells were incubated with nanoparticle fucoidan or native fucoidan (1 and 2 mg/mL) for 72 h. Cells were stained with Apo2.7 and analyzed by flow cytometry. The data shown here are from a representative experiment repeated 3 times with similar results. Ab, antibody; Nano-Fuc, nanoparticle fucoidan; Native-Fuc, native fucoidan; (**D**) Effect of Z-VAD-FMK, a pan-caspase inhibitor, on nanoparticle fucoidan-induced apoptosis. 143B cells were pre-treated for 1 h with 20 µM of Z-VAD-FMK before the addition of nanoparticle fucoidan. Cell viability was analyzed 72 h later using the water-soluble tetrazolium (WST)-8 assay. Data represent the percentage of cell survival compared with the control and are expressed as mean ± SD (*n* = 3).

In the initial set of experiments, the cytotoxic effect of different concentrations of nanoparticle fucoidan on a human osteosarcoma cell line 143B was evaluated using water-soluble tetrazolium (WST)-8 assays. Nanoparticle fucoidan reduced the viability of these cells in dose- and time-dependent manners (Figure 2A). The maximum decline in cell viability of 80% after 72 h culture was achieved with nanoparticle fucoidan concentration of 2 mg/mL, beyond which the effect plateaued. Thus, in all subsequent studies, we used nanoparticle fucoidan at concentrations of 1 and 2 mg/mL.

Apo2.7 specifically detects the 38 kDa mitochondrial membrane antigen 7A6 expressed on the mitochondrial outer membrane during apoptosis [15]. Nanoparticle fucoidan induced apoptosis in 143B cells, as shown by Apo2.7 staining, in dose-and time-dependent manners (Figure 2B). After incubation for 72 h, 35% and 73% of cells treated with 1 or 2 mg/mL nanoparticle fucoidan stained positively for Apo2.7, respectively, compared with only 15% of cells treated with the medium alone (Figure 2B).

Next, we investigated the effect of HMWF on the induction of apoptosis. The apoptosis-inducing activity of native fucoidan (molecular weight: 80 kDa) was lower (25%) than that of nanoparticle fucoidan (94%) when both were used at 2 mg/mL (Figure 2C). These results suggest that nanoparticle fucoidan is more active than native fucoidan in the induction of apoptosis of cultured osteosarcoma cells.

Caspases are a family of cysteine acid proteases that play an integral role in the cascade that leads to apoptosis [16]. Pre-incubation of 143B cells with the pan-caspase inhibitor Z-VAD-FMK 60 min before the addition of nanoparticle fucoidan significantly inhibited the decrease in cell viability (Figure 2D). These results indicate that nanoparticle fucoidan induces apoptosis through the activation of caspase pathway.

### 2.2. Effects of Fucoidan on Tumorigenesis of Osteosarcoma Cells in Mice

**Figure 3:**
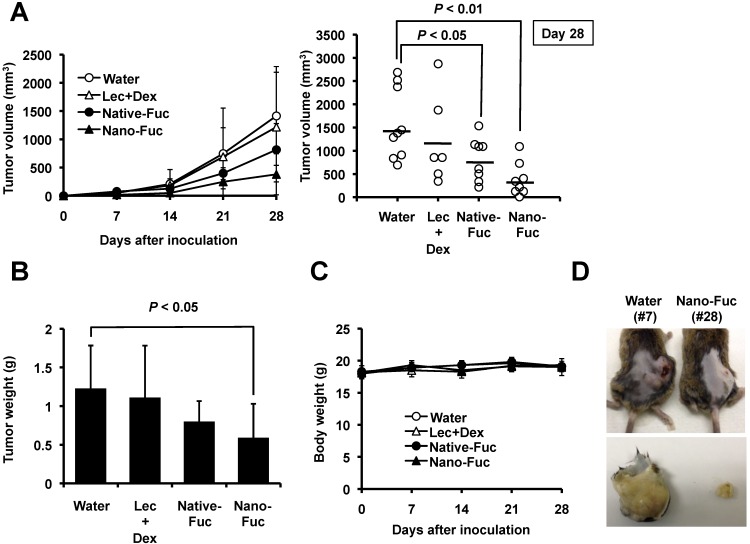
Effects of fucoidan on tumor growth in mice xenografted with LM8 osteosarcoma cells. Water (*n* = 9), lecithin and dextrin (Lec + Dex) (*n* = 6), native fucoidan (Native-Fuc) (*n* = 8) or nanoparticle fucoidan (Nano-Fuc) (*n* = 8) were administrated orally every day for four weeks, (**A**) Effects of fucoidan on tumor volume; (**B**) Effects of fucoidan on tumor tissue weight; (**C**) Effects of fucoidan on body weight. Data are mean ± SD (*n* = 3); (**D**) Photographs of nanoparticle fucoidan-treated and untreated osteosarcoma-bearing mice four weeks after subcutaneous inoculation with LM8 (top). Tumors were excised on day 28. The photographs show representative tumor of an untreated mouse (bottom left) and that of nanoparticle fucoidan-treated mouse (bottom right).

To examine the potential use of fucoidan in the treatment of osteosarcoma, we tested it’s effect on tumor growth *in vivo*. After inoculation of murine osteosarcoma LM8 tumor cells in the back of C3H mice, the animals were treated with oral 100 mg/kg/day nanoparticle fucoidan or native fucoidan. Mice of the control group were treated with water (vehicle) only. Treatment with native fucoidan (*P* < 0.05) and nanoparticle fucoidan (*P* < 0.01) resulted in significant reduction in tumor volume in the LM8 xenografted mice, relative to the control (Figure 3A,D). In the next set of experiments, we demonstrated that the anti-sarcoma effect was not due to significant changes in lecithin and dextrin used for liposome preparation (Figure 3A). Relative to the control, treatment with native fucoidan and nanoparticle fucoidan resulted in reduction of tumor tissue weight, though the effect of the latter was significant (*P* < 0.05) compared with marginal significance for the former (*P* = 0.065) (Figure 3B). Importantly, both preparations did not induce significant reduction in body weight throughout the experimental period, compared with the control (Figure 3C). With regard to side effects of the drug, no gross abnormalities were noted in mice treated at the selected dose.

### 2.3. Fucoidan Induces Apoptosis in Osteosarcoma Xenograft Model

**Figure 4:**
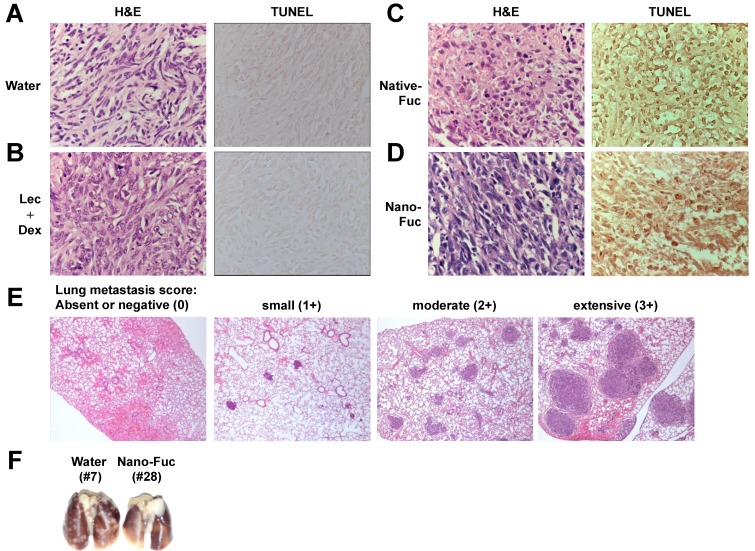
Fucoidan induces apoptosis in local primary tumors and inhibits lung metastases in mice, (**A**–**D**) hematoxylin and eosin (H&E) staining and *in situ* terminal deoxynucleotidyl transferase-mediated dUTP nick end labeling (TUNEL) assays of LM8-derived tumor tissues. Tumors obtained from mice treated with water (**A**), lecithin and dextrin (Lec + Dex) (**B**), native fucoidan (Native-Fuc) (**C**) or nanoparticle fucoidan (Nano-Fuc) (**D**) daily were harvested on day 28 and subjected to analysis. Magnification, ×400; (**E**) Microphotographs of the lungs in treated mice. A four-point scale was used for quantitative scoring of invasion of LM8 cells into the lung, where 0 (absent or negative) indicates no invasion, and 1+, 2+ and 3+ indicate small, moderate and extensive invasion, respectively. Magnification, ×25; (**F**) Representative pictures showing gross appearance of the lungs of mice treated (right) or untreated (left) with nanoparticle fucoidan examined on day 28.

Hematoxylin and eosin (H&E) staining and terminal deoxynucleotidyl transferase-mediated dUTP nick end labeling (TUNEL) assay were performed to evaluate the cell death mechanism *in vivo*. Figure 4A,B shows dark blue hematoxylin-stained nuclei and pink eosin-stained cytoplasm, whereas native fucoidan- and nanoparticle fucoidan-treated tumors (Figure 4C,D, H&E) show large numbers of apoptotic cells with nuclear condensation and fragmentation. Correspondingly, the density of viable tumor cells was substantially decreased. TUNEL staining confirmed more extensive apoptosis in the native fucoidan- and nanoparticle fucoidan-treated tumors compared with the control with LM8-derived tumors (Figure 4C,D, TUNEL).

### 2.4. Fucoidan Inhibits Metastasis of Mouse Osteosarcoma Cells to the Lung

In this model, subcutaneous inoculation of LM8 cells in the back of C3H mice resulted in spontaneous metastasis to the lung in addition to the formation of local primary tumors. The antimetastatic activity of fucoidan was also found when spontaneous metastasis of LM8 cells to the lung was compared in tumor-bearing, fucoidan-treated mice and in water-treated control mice. A four-point scale was used for quantitative scoring of the invasion of LM8 cells into the lung, where 0 (absent or negative) indicates no invasion, and 1+, 2+ and 3+ indicate small, moderate and extensive invasion, respectively. The anatomic levels of metastatic nodules on the lung surface correlated with microscopic evaluation (Figure 4E,F). All nine control mice inoculated with LM8 cells developed spontaneous lung metastases with a mean score of 1.89 ± 0.78. In contrast, visible gross lung nodules were not found in 3/8 mice treated with native fucoidan and nanoparticle fucoidan. The mean scores of mice treated with native fucoidan and nanoparticle fucoidan were 0.88 ± 0.83 and 1.13 ± 1.13, respectively. Thus, oral administration of native fucoidan and nanoparticle fucoidan decreased the score compared with that observed in the control group, and there was a significant difference between the native fucoidan group and the control group (*P* < 0.05). Five of the six mice treated with lecithin and dextrin developed spontaneous metastasis to the lung with a mean score of 1.50 ± 1.05. Collectively, treatment with native fucoidan and nanoparticle fucoidan suppressed pulmonary metastases.

### 2.5. Cell Permeation of Fucoidan

Finally, Caco-2 cell transport studies were performed on native fucoidan and nanoparticle fucoidan. As shown in Table 1, the amount of permeated nanoparticle fucoidan and permeation rate of nanoparticle fucoidan were significantly higher than native fucoidan.

**Table 1:** Permeability of Caco-2 cell monolayers to fucoidan.

Sample	Amount of Permeated Fucoidan (µg/cm^2^/h)	Permeation Rate of Fucoidan (%)
Native fucoidan	0.034 ± 0.03	0.001 ± 0.001
Nanoparticle fucoidan	5.298 ± 0.341	0.229 ± 0.015

## 3. Experimental Section

### 3.1. Cell Lines and Reagents

Human osteosarcoma cell line, 143B, and mouse osteosarcoma cell line, LM8, were maintained in RPMI 1640 and Eagle’s Minimal Essential Medium supplemented with 10% heat inactivated fetal bovine serum, penicillin (50 U/mL) and streptomycin (50 µg/mL) in a humidified incubator, respectively. The human colon adenocarcinoma cell line, Caco-2, was purchased from Cell Bank, RIKEN BioResource Center (Tsukuba, Japan). For cell culture, α-Minimum Essential Medium was supplemented with 1% non-essential amino acids, 20% heat inactivated fetal bovine serum, penicillin (100 U/mL) and streptomycin (100 µg/mL). A caspase inhibitor, Z-VAD-FMK, was purchased from Promega (Madison, WI, USA).

### 3.2. Preparation of Native Fucoidan from Seaweed

The brown seaweed *Cladosiphon okamuranus* Tokida cultivated in Okinawa, Japan, was suspended in water, 1.13% (w/vol) citric acid was added to the solution, and then heated at 90 °C for 40 min. The suspension was neutralized with NaOH solution, then centrifuged at 3500 rpm by decantation centrifugal separator. The supernatant was collected, filtered using Cohlo filter and concentrated by ultrafiltration (molecular weight cutoff 6000). The extracts were dried by spraydrier and the molecular weight of this native fucoidan was 80 kDa. Soybean lecithin, SLP-PC70 (Tsuji Oil Mill Co., Matsusaka, Japan), and dextrin (Pinedex, Matsutani Chemical Industry Co., Itami, Japan) were added as control for nanoparticle fucoidan, and this mixture was used as native fucoidan. Native fucoidan was dissolved in RPMI 1640 before *in vitro* experiments or distilled water before *in vivo* experiments.

### 3.3. Preparation of Liposome Encapsulating Fucoidan

Native fucoidan was hydrolyzed to produce LMWF (molecular weight 2–10 kDa). The hydrolysis process was carried out by dissolving native fucoidan into 2.0% (w/vol) citric acid mixture at 100 °C for 24 h. After neutralization with NaOH solution, the suspension was centrifuged at 3500 rpm by decantation centrifugal separator. The supernatant was collected and filtered. The liposome fraction was prepared by the mechanochemical method [17] using the following procedure. The hydrolyzed fucoidan extract and soybean lecithin, SLP-PC70 solution, were mixed well. These solutions were dispersed by a homogenizer (TK HOMO MIXER MARK II, PRIMIX, Osaka, Japan) at a revolving rate of 8000 rpm for 10 min. The resulting solution was then treated once with the microfluidizer (M110-E/H, MIZUHO Industrial Co., Osaka, Japan). The operation was carried out with an inlet pressure of 100 MPa. Dextrin was then added and mixed. The liposome encapsulating LMWF was dried by spraydrier for subsequent use. FFEM was used to determine the structure of lecithin (SLP-PC70) liposomes, as described previously [17].

### 3.4. Assessment of Cell Viability and Apoptosis

Cell viability was determined by color reaction with WST-8 (Wako Pure Chemical Industries, Osaka, Japan). Basically, mitochondrial dehydrogenase cleavage of WST-8 to formazan dye provided a measure of cell viability. Briefly, 1 × 10^5^ cells/mL were incubated in triplicate in a 96-well microculture plate in the presence of different concentrations of fucoidan for 24–72 h. Subsequently, WST-8 was added to each well. After 4 h of additional incubation, absorption values at 450 nm were determined with an automatic microplate reader. Values were normalized to untreated control samples. Apoptotic cells were detected by staining with phycoerythrin-conjugated APO2.7 antibody (Beckman Coulter, Marseille, France), which specifically detects the 38 kDa mitochondrial membrane antigen 7A6 [15], followed by analysis by flow cytometry.

### 3.5. Preparation of Sarcoma Animal Model

LM8 cells (5 × 10^6^ cells/mouse) in 0.1 mL phosphate buffered saline were injected subcutaneously into the back of five-week-old female C3H mice obtained from Japan SLC (Hamamatsu, Japan) on day 0. Treatment was initiated one day after cell injection. Groups of 6–9 mice were used to generate LM8 tumors, and each group was treated with water, lecithin and dextrin, native fucoidan or nanoparticle fucoidan. For the latter, lecithin and dextrin were used as a control. Lecithin, dextrin, native fucoidan and nanoparticle fucoidan were dissolved in distilled water, and 100 mg/kg body weight of fucoidan or vehicle only was administered by oral gavage every day for 28 days. The mice were weighed once a week and tumor diameters were measured weekly. The mice were monitored daily for evidence of morbidity including anorexia, dehydration, dyspnea, decreased activity and grooming behavior. On day 28, all mice were euthanized and autopsied to confirm metastatic lung disease. Lung infiltration by LM8 cells was evaluated by H&E staining. Primary tumors were dissected out and weighed, then processed for staining with H&E and TUNEL using a commercial kit (Roche Applied Science, Mannheim, Germany). This experiment was performed according to the guidelines for Animal Experimentation of the University of the Ryukyus and approved by the Animal Care and Use Committee of the same University.

### 3.6. Permeation across Caco-2 Monolayers

Caco-2 cells were seeded at a density of 30,000 cells onto 24-well Transwell plates with an insert area of 0.33 cm^2^ and a pore size of the polycarbonate membrane of 0.4 µm. The culture medium was changed every 48 h for the first 10 days and every 24 h thereafter, and cell monolayers were used between 19 and 20 days post-seeding. The transepithelial electrical resistance of cultured cells on Transwell inserts was measured before and after each permeation experiment with a Millicell-Electorical Resistance System ohmmeter (Millipore, Bedford, MA, USA). Physiologically and morphologically well-developed confluent Caco-2 monolayers (at least 19-day-old) with transepithelial electrical resistance values typically above 250 Ωcm^2^ were used in the experiments. The apical side of Caco-2 cell monolayers cultured in 24-well Transwell inserts was washed twice with phosphate-buffered saline. Fucoidan was added to the apical compartment with respect to the cell monolayer which was then incubated at 37 °C for 2 h. Permeation of fucoidan across the cell monolayer was measured by sampling the solutions in both compartments after 2 h of incubation with a sample. The obtained samples were evaporated to dryness and the resulting residue was dissolved in 10 µL of distilled water. Then, 10 µL of 8 M trifluoroacetic acid solution was added to the sample solution, and the solution was incubated at 100 °C for 3 h. After cooling to room temperature, the reaction mixture was evaporated to dryness. The resulting residue was dissolved in 40 µL of 2-propanol and again evaporated to dryness. Next, 10 µL of distilled water and 40 µL of the 4-aminobenzoic acid ethyl ester reagent solution were added to the residue, and the mixture was incubated at 80 °C for 1 h. Afterwards, the mixture was cooled to room temperature, and 0.2 mL of distilled water and an equal volume of chloroform were added. After vigorous vortexing, the mixture was centrifuged and the upper aqueous layer was subjected to HPLC LC-2000Plus (JASCO, Tokyo, Japan) to determine fucose concentration in the samples.

### 3.7. Statistical Analysis

All values are expressed as mean ± SD. Differences between groups were analyzed for statistical significance by the unpaired Student’s *t*-test. A confidence level of *P* < 0.05 was chosen as indication of statistical difference.

## 4. Conclusions

We have recently prepared liposome-encapsulated fucoidan for nano-formulation. In this study, we examined the anti-neoplastic activity of nanoparticle fucoidan against osteosarcoma cell lines. Our examination using viability and apoptosis assays demonstrated that nanoparticle fucoidan induced apoptosis of 143B cells and that this action was mediated through the caspase pathway. Nanoparticle fucoidan was more active than native fucoidan in inducing apoptosis *in vitro*. Furthermore, both nanoparticle fucoidan and native fucoidan demonstrated significant inhibition of tumor growth and spontaneous metastases in the lung from LM8 mice tumor xenografts. The tumor volume and weight were decreased in nanoparticle fucoidan group compared with those observed in native fucoidan group. However, the lung metastasis score was decreased in native fucoidan compared with that observed in nanoparticle fucoidan. One limitation of this study was that we did not use non-encapsulated LMWF as a control to study the effect of nanoparticles on cells and in mice. In other words, the present study did not examine the effects of molecular weight condition on anti-osteosarcoma activity of fucoidan. The sensitive sandwich ELISA method for the measurement of serum fucoidan was recently developed [18], although detection of fucoidan in the serum has been thought to be impossible. The measurement of fucoidan in blood, tissues and organs may be useful to evaluate its beneficial effects *in vivo*. The findings that fucoidan hindered osteosarcoma LM8 tumor growth, and reduced tumor mass and lung metastases *in vivo* suggest a potential therapeutic application. Furthermore, the results demonstrate that encapsulation of fucoidan into nanoparticles enhances its anti-osteosarcoma effects, at least in part by increased permeability.
